# Supplementary material for: Associations between Plasma Folate and Vitamin B12, Blood Lead, and Bone Mineral Density among Adults and Elderly Who Received a Health Examination
Source: Nutrients. 2022 Feb 21;14(4):911. doi: 10.3390/nu14040911 (PMC8878955; doi:10.3390/nu14040911)
Supplement: Supplementary file 1 [file nutrients-14-00911-s001.zip › nutrients-1605312-supplementary.pdf]

Supplementary Tables

Table S1. The validity and reliability of the measurement of urinary arsenic species, red blood cell lead and cadmium, and plasma homocysteine, vitamin B_{12}, and folate.

Measurements	Instrument	Detection limit	Recovery rate (%)	Standard reference materials (SRM)	Coefficient of variance (%)
Arsenite (As^{III})	High-performance liquid chromatography-hydride generator-atomic absorption spectrometry	0.02 µg/L	93.8 - 102.2	SRM 2670 (National Institute of Standards and Technology, Gaithersburg, MD) certificate value 480 ± 100 µg/L inorganic arsenic, in our system 507 ± 17 µg/L (n = 4).	< 10
Arsenate (As^{V})		0.10 µg/L			
Monomethylarsonic acid (MMA^{V})		0.07 µg/L			
Dimethylarsinic acid (DMA^{V})		0.06 µg/L			
Blood lead	Inductively coupled plasma mass spectrometry	0.32 µg/dL	100 ± 20	SRMs (Seronorm Trace Elements Whole Blood L-2 (Lot 1103129)) certificate value 5.8 µg/L (range: 5.4–6.2 µg/L) in our system 6.1 ± 0.5 µg/L	< 10
Blood cadmium	Inductively coupled plasma mass spectrometry	0.07 µg/L	100 ± 20	SRMs (Seronorm Trace Elements Whole Blood L-2 (Lot 1103129)) certificate value 310.0 µg/L (range 186.0–434.0 µg/L), in our system 329.0 ± 17.0 µg/L	< 10
Plasma Vitamin B_{12}	Radioassay kit (Bio-Rad, Richmond, CA) and the 1470 Wizard series gamma counter	75 pg/mL			6.8
Plasma folate	Radioassay kit (Bio-Rad, Richmond, CA) and the 1470 Wizard series gamma counter	0.6 ng/mL			9.3
